# Engineered xCas9 and SpCas9‐NG variants broaden PAM recognition sites to generate mutations in *Arabidopsis* plants

**DOI:** 10.1111/pbi.13148

**Published:** 2019-05-29

**Authors:** Zengxiang Ge, Leiqi Zheng, Yuling Zhao, Jiahao Jiang, Emily J. Zhang, Tianxu Liu, Hongya Gu, Li‐Jia Qu

**Affiliations:** ^1^ State Key Laboratory for Protein and Plant Gene Research Peking‐Tsinghua Center for Life Sciences at College of Life Sciences Peking University Beijing China; ^2^ Aragon High School San Mateo CA USA; ^3^ The National Plant Gene Research Center (Beijing) Beijing China

**Keywords:** genome editing, xCas9, SpCas9‐NG, NG PAMs, Arabidopsis

The clustered regularly interspaced short palindromic repeat (CRISPR)‐associated protein 9 (Cas9) system has been widely used for genome modification in various species, including plants (Hsu *et al*., 2013; Wang *et al*., 2015). This customized endonuclease system includes two components: a single guide RNA (sgRNA) consisting of an artificial fusion of a crRNA and a tracrRNA for target recognition and the Cas9 endonuclease for targeted DNA cleavage. A specific protospacer adjacent motif (PAM) near the target site is essential for endonuclease recognition. For example, the function of the most robust and widely used *Streptococcus pyogenes* Cas9 (spCas9) is mostly dependent on the canonical PAM sequence NGG, although it can recognize other non‐canonical NAG and NGA PAMs to a lesser extent (Hsu *et al*., 2013). Therefore, the number of targetable genomic loci is largely restricted. To relieve this restriction, several other Cas9 effector proteins and Cas9 variants with distinct PAM specificities have been developed, such as Cpf1 that recognizes T‐rich PAM (Zetsche *et al*., 2015). Recently, two newly engineered SpCas9 variants, xCas9 and SpCas9‐NG, have been reported to recognize relaxed NG PAMs and induce indels at endogenous target sites in mammalian cells (Hu *et al*., 2018; Nishimasu *et al*., 2018). However, whether these newly engineered SpCas9 variants function well in the dicotyledonous model plant Arabidopsis has not yet been reported. In this study, we examined the activities of xCas9 and SpCas9‐NG in transgenic Arabidopsis plants and demonstrated that these two variants could, to a certain degree, expand the use of CRISPR *in planta*, although with a reduced efficiency. Both variants induced effective mutation types, including homozygous/biallelic and heterozygous mutations, which would generate loss‐of‐function mutants in the Arabidopsis T1 generation.

In a first attempt, we introduced the corresponding mutations into the widely used ZmCas9 (*Zea mays* codon‐optimized Cas9) in Arabidopsis (Wang *et al*., 2015), generating the xCas9 3.7 variant with A262T/R324L/S409I/E480K/E543D/M694I/E1219V mutations (Hu *et al*., 2018) (Figure 1a). Given that xCas9 was able to recognize NG PAMs (Hu *et al*., 2018), we designed different targets harbouring NGG, NGA, NGC or NGT PAMs, all of which could specifically target the Arabidopsis *FERONIA* (*FER*) gene. Loss‐of‐function mutants of this gene show identical and distinct phenotypes in seedling and leaf development (Duan *et al*., 2010) (Figure 1b). The final all‐in‐one vector, containing two independent SgRNAs of each PAM under the *AtU6* promoter and Cas9 (wild‐type ZmCas9 or xCas9 3.7) driven by the optimized *EC1* promoter, was constructed as reported (Wang *et al*., 2015) and transformed into *Arabidopsis thaliana* (Col‐0) by Agrobacterium‐mediated transformation (Figure 1a). All the transgenic plants, generated from the ZmCas9 CRISPR system and ZmCas9‐mutated CRISPR system, were grown at 22 ± 2 °C under long‐day conditions (16 h light/8 h dark cycles).

**Figure 1:**
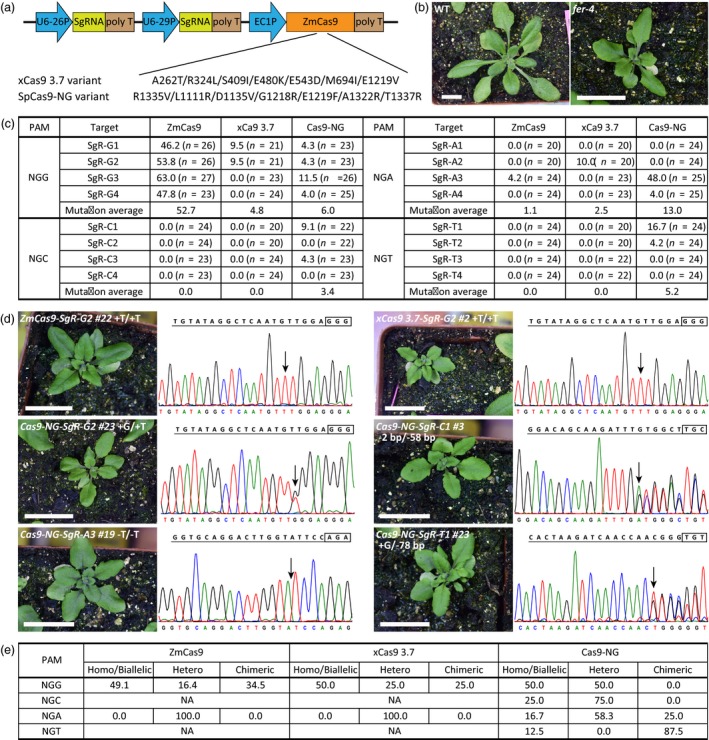
Gene editing by xCas9 3.7 and Cas9‐NG variants in Arabidopsis. (a) The all‐in‐one expression vectors for Cas9 (wild‐type ZmCas9 or Cas9 variants) and SgRNAs in Arabidopsis. U6‐26P and U6‐29P, two *U6* promoters; EC1P, optimized *EC1* promoter; and ZmCas9, *Zea mays* codon‐optimized Cas9. (b) Phenotype of WT and *fer‐4* mutant. The pictures of the plants were taken 3 weeks after being transplanted to soil. Scale bar, 1.0 cm. (c) Frequencies of targeted gene mutations induced by Cas9 variants. Mutation frequency (%) is calculated from the number of mutated plants confirmed by direct sequencing in all the transgenic plants. (d) Phenotypes and genotypes of the *fer‐4‐*like T1 transgenic lines induced by CRISPR. The pictures of the plant were taken 3 weeks after being transplanted to soil. Scale bar, 1.0 cm. The SgRNAs and the PAMs are highlighted with underline and frame. Sequencing chromatogram of each line is shown under the SgRNA sequence, and arrow points to the position with mutation. Mutations of Cas9‐NG‐SgR‐C1 #3 and Cas9‐NG‐SgR‐T1 #23 were analysed using DSDecodeM. (e) The average frequencies of each zygotic type induced by Cas9 variants. NA, no mutations available. Homo/Hetero, homozygous/heterozygous.

We then extracted the genomic DNA from the seedling or young leaf cells of the hygromycin B‐resistant T1 transgenic plants, amplified the DNA fragments flanking the target sites by PCR and conducted Sanger DNA sequencing analysis to determine whether wild‐type ZmCas9 or xCas9 3.7 variant could induce mutations or not. The sequencing results were analysed by using MEGA X (https://www.megasoftware.net/), Chromas (http://technelysium.com.au/wp/chromas/) and DSDecodeM (http://skl.scau.edu.cn/dsdecode/). The mutation frequency (%) was calculated from the number of mutant plants among all the transgenic plants based on the sequencing results. As a positive control, ZmCas9 was able to target the canonical NGG PAM with a high efficiency (from 46.2% to 63.0%, Figure 1c). However, with the same SgRNAs, we found that <9.5% of the transgenic lines were successfully edited by xCas9 3.7. Specifically, the mutation efficiencies of ZmCas9 for SgR‐G1 and SgR‐G2 were 46.2% and 53.8%, respectively, whereas those of the xCas9 3.7 targets were only 9.5%. ZmCas9 targeted SgR‐G3 or SgR‐G4 with an efficiency as high as 47.8% but xCas9 3.7 failed to edit these two targets (Figure 1c). These results indicate that xCas9 3.7 can recognize some NGG sequences, but with much lower efficiency. For NGA, NGC or NGT PAMs, the editing efficiency of ZmCas9 was greatly reduced (0%–4.2%, Figure 1c). Similarly, only 10.0% of the transgenic lines harbouring NGA PAM (i.e. SgR‐A2 target) were edited by xCas9 3.7, with no mutations of NGC and NGT PAMs as well other types of NGA PAMs (Figure 1c). These results suggest that xCas9 3.7 can barely edit genomic DNA at these non‐NGG PAMs with an efficiency comparable to that of wild‐type ZmCas9.

To test the activity of the SpCas9‐NG variant *in planta*, we adopted the same strategy and analysed the transgenic plants of the ZmCas9 CRISPR system or SpCas9‐NG CRISPR system under the same conditions. We found that SpCas9‐NG was capable of recognizing NGG PAMs with the mutation ratio as high as 11.5%, which was much lower than that of ZmCas9 for the same SgRNA (Figure 1c). Importantly, we found that SpCas9‐NG could target other types of PAMs, with targeting efficiencies of 3.4% for NGC, 13.0% for NGA and 5.2% for NGT on average, which were higher than these of ZmCas9 and xCas9 3.7 at these target sites (Figure 1c). Moreover, we noticed a very high targeting efficiency of SgR‐A3 (NGA PAM) by the SpCas9‐NG variant (i.e. 48.0%), which was comparable to that of wild‐type Cas9 targeting NGG PAMs. Although not all the NGA PAMs could be efficiently targeted by SpCas9‐NG, these findings also provide a hint that there is some space to improve the application of SpCas9‐NG at the NGA site. Therefore, our results show that SpCas9‐NG variant can induce indels at non‐NGG sites with higher efficiency than ZmCas9 or xCas9 3.7, but not as efficiently as ZmCas9 for NGG PAM in Arabidopsis.

Although xCas9 3.7 and SpCas9‐NG exhibited less activities for genome‐editing *in planta*, we were still able to generate some mutant lines for the phenotypic analysis in the T1 generation. As shown in Figure 1d, several lines, such as xCas9 3.7 for SgR‐G2, Cas9‐NG for SgR‐C1 and Cas9‐NG for SgR‐A3, displayed a significantly reduced stature, which were consistent with the *fer*‐*4* mutant (Figure 1b, d). After genotyping these *fer‐4*‐like plants by sequencing the region around the target sites, we confirmed that they were indeed loss‐of‐function mutants with homozygous/biallelic mutations (Figure 1d). We also found that these types of homozygous/biallelic mutations as well as the heterozygous mutations predominated among the mutations induced by xCas9 3.7 and SpCas9‐NG for broad PAMs, similar to the situation of ZmCas9 for NGG PAMs (Figure 1e). Thus, we demonstrated that both the xCas9 3.7 and SpCas9‐NG variants could be used to generate certain mutation types for the candidate genes in Arabidopsis as an alternative to the wild‐type Cas9, especially when the NGG PAMs in these genes are limited.

Taken together, the results of this study expand the application of engineered Cas9 variants that recognize non‐NGG PAMs in Arabidopsis *in planta*. Similar results have recently been reported in rice while this paper is being reviewed, in which xCas9 and SpCas9‐NG variants successfully recognize non‐canonical PAMs and induce mutations, with reduced efficiency than wild‐type Cas9 on NGG PAMs (Hua *et al*., 2019; Ren *et al*., 2019; Wang *et al*., 2018; Zhong *et al*., 2019). We propose that this phenomenon is a consequence of the Agrobacterium‐mediated transformation method used in the plants, especially in Arabidopsis, which is quite different from the transfection methods used for mammalian cell transformation, in which *in vitro*‐transcribed Cas9‐encoding mRNA and SgRNAs are coinjected (Nishimasu *et al*., 2018; Wang *et al*., 2018). Thus, these two Cas9 variants may not function as well in transgenic plants as in mammals. Moreover, it is worth noting that SpCas9‐NG functions more efficiently at non‐NGG PAMs than wild‐type Cas9 and xCas9 3.7 in Arabidopsis, suggesting more expansive possibilities to improve the targeting space based on the SpCas9‐NG variant in Arabidopsis. Regardless, we also found that both xCas9 3.7 and SpCas9‐NG can successfully induce homozygous/biallelic and heterozygous mutations at broad PAM targets once the plants are edited by these systems. Therefore, we propose that these variants could be used as an alternative to wild‐type Cas9 in Arabidopsis to generate certain types of mutations for candidate genes when NGG PAMs are not available.

## Conflict of interest

The authors declare no conflict of interest.

## Author contributions

L.‐J.Q. and Z.G. designed the studies. Z.G. and L.Z. performed experiments. Y.Z., J.J., E.Z. and T.L. coordinated the generation of transgenic plants. L.‐J.Q, Z.G. and L.Z. wrote the paper with input from H.G.
